# Adaptation and Implementation of Self-System Therapy for Older Adults with Advanced Lung Cancer: Pilot Trial Results

**DOI:** 10.3390/cancers17172809

**Published:** 2025-08-28

**Authors:** Katherine Ramos, Aliza Ayaz, Jennie Riley, Kaylee Faircloth, Laura S. Porter, Timothy J. Strauman

**Affiliations:** 1 Department of Psychiatry & Behavioral Sciences, Duke University Medical Center, Durham, NC 27710, USAlaura.porter@duke.edu (L.S.P.);; 2 Department of Population Health Sciences, Duke University Medical Center, Durham, NC 27710, USA; 3 Department of Medicine, Duke University Medical Center, Durham, NC 27710, USA; 4 Department of Psychology and Neuroscience, Duke University, Durham, NC 27708, USA; aliza.ayaz@duke.edu

**Keywords:** advanced lung cancer, older adults, self-system therapy, psychosocial intervention, psychological distress, pilot trial, feasibility, acceptability, palliative psychology, quality of life

## Abstract

**Background/Objectives:** Advanced lung cancer is a highly distressing disease that negatively impacts older adults. Supportive care interventions designed for this population are scarce and often inaccessible due to competing demands and transportation access. We adapted and refined an evidence-based treatment, Self-System Therapy (SST), to address the unmet needs of older adults with advanced cancer. **Methods:** Guided by principles of implementation science, we conducted patient interviews, focus groups, and user testing to refine our new SST for the lung cancer (SST-LC) protocol. We then conducted a single-arm pilot trial (clinicaltrials.gov NCT04057196) for patients aged 65+ and above with Stage III or IV lung cancer (N = 30). Benchmarks for acceptability, feasibility, and preliminary changes in outcome measures were assessed. **Results:** Our study met the desired recruitment goals and demonstrated high treatment adherence rates (89%) and satisfaction rates (85%), indicating that SST-LC was feasible and well-received. Participants also showed reductions in distress and depression, and improvements in emotional and functional well-being from baseline to post-intervention, with effects mostly maintained at follow-up. Physical well-being, social well-being, and quality of life showed smaller, non-significant changes. Feedback from participants also suggested that SST enhanced their resilience and ability to cope with cancer-related challenges, but also indicated a preference for fewer sessions. **Conclusions:** SST for older adults living with advanced lung cancer is feasible and acceptable. Moreover, this supportive care intervention shows promise in addressing psychological distress, emotional well-being, and functional well-being in older adults. Future research will include testing the efficacy of SST in a larger randomized controlled trial.

## Simple Summary

Lung cancer is a highly prevalent disease that disproportionately affects adults aged 65 and older. Existing interventions addressing the psychological burden of lung cancer are frequently inaccessible and inadequately tailored to this population’s unique needs. The aim of this pilot trial was to assess the feasibility, acceptability, and *preliminary signal* of Self-System Therapy (SST) for distress reduction in advanced lung cancer. Findings from this study may inform future interventions and supportive care strategies for older adults living with advanced cancer by fostering resilience, reducing distress, and improving quality of life.

## 1. Introduction

Cancer is a common and chronic condition that presents an inequitable burden of disease. More than half of individuals diagnosed with cancer are 65 and older, and by 2030, it is projected that 70% of all cancers will be diagnosed in this demographic [1,2]. Among this age cohort, lung cancer is the most diagnosed cancer (close to 70%) [3,4,5,6]. Lung cancer is also reported as the most psychologically distressing type of cancer compared to other cancer types [7,8]. Distressing symptoms, such as elevated levels of depression and anxiety, are commonly experienced. These symptoms are often exacerbated by impaired functioning and extensive physical symptomatology. Meta-analytic evidence suggests that behaviorally based interventions targeting emotional distress and promoting engagement in physical activity may yield positive outcomes for cancer patients, including improved mood and functioning [9,10,11].

Despite the potential benefits that older adults with advanced lung cancer may experience from behavioral interventions, there are substantial challenges in adapting such interventions for this population. Current supportive-care interventions often neglect normative aging concerns that address identity, role transitions, and changing goal structures amid physical health challenges [12,13,14]. Notably, briefer interventions (e.g., from traditional 12–16 weekly sessions reduced to 8 sessions or less) may prove to be more tolerable and less burdensome [15,16,17], as these individuals are continually experiencing rapidly changing physical health, coupled with the real threat of having a minimal amount of time to live. Thus, brief, efficacious, and accessible psychological support may offer older adults a faster “turnaround” to utilize newly learned coping strategies in daily living and enhance their quality of life.

To provide support for this population, in this study, we adapted and implemented a psychosocial behavioral intervention, Self-System Therapy (SST) [18,19], to target both physical and psychological well-being. SST is an empirically validated intervention grounded in current models of motivation and goal pursuit, that has shown efficacy in reducing distress among depressed adults [19]. SST can be summarized in four questions: What are your promotion (approach) and prevention (avoidance) goals? What are you doing to try to attain them? What is keeping you from making progress? What can you do differently? The goals of SST for lung cancer (SST-LC) are to target cancer-related distress and to enhance motivation by supporting more involvement in physical activity (linked to personal values) that offers older adults a sense of purpose and meaning in their daily lives. This paper presents the findings of our study, guided by a framework known as the Adaptome within implementation science [20], which encompasses key features of intervention refinement, such as modifying service settings, target audiences, and models of delivery, all delivered via telehealth.

### Framework of Self-System Therapy: Intervention and Refinement

The adaptome framework [20] guided our adaptation of Self-System Therapy [18,19]. Adaptome takes into account that changing contexts, as well as differences in populations, can impact and influence the delivery, implementation, and refinement of evidence-based treatment interventions [20]. We leveraged this particular framework within implementation science to support the adaptation of SST [18,19] to meet the needs of older adults with advanced lung cancer. In particular, we focused on the necessary modifications (e.g., focusing on cancer), delivery type (e.g., delivering the intervention through Zoom [21]), and session length (e.g., refining content to eliminate duplication). We used the adaptome framework [20] as a guide to structure interviews with a total of 11 older adults with lung cancer, aiming to inform the refinement of the SST intervention. Each participant was reimbursed USD 75. Interviews were primarily conducted as three focus groups (with a total of 8 participants). For participants who could not attend the scheduled focus groups due to illness, or arrived late to join the session, we offered individual interviews. We used this approach with a total of 3 participants. During interviews and focus groups, session content we reviewed focused on the impact of the cancer experience, coping with illness, and adopting behaviors that promote health and prevent maladaptive health behaviors (e.g., isolating or not exercising due to fears of dyspnea). We also discussed the number of proposed sessions for delivering SST (up to 12) including delivering the intervention individually or as a group. Participants endorsed that up to 12 sessions may seem sufficient and that individual sessions may offer a more tailored experience. Following the completion of these interviews, and gathered feedback, we conducted user testing of the developed SST for lung cancer (SST-LC) protocol with 5 participants. Participants completed the SST-LC protocol individually via telehealth (e.g., Zoom [21]). They provided feedback after each session on the feasibility of learning and applying the techniques and skills, the perceived usefulness of each skill, the use of videoconferencing as the mode of delivery, and general feedback. Finalized session content is described in Section 2.3.

Feedback during the user testing phase was used to make final revisions to the SST-LC protocol before beginning the pilot trial. Each participant in user testing was also reimbursed USD 75. We presented a summary of participant feedback to the whole study team and made decisions about any necessary adjustments to the SST-LC protocol before initiating the pilot trial. The adjustments we made included keeping sessions between 8 and 12 sessions, limiting session length to an hour, and de-emphasizing content focused solely on the experience of depression to place greater emphasis on psychological distress (more broadly), which participants identified as more relevant to their experience with cancer. In preparation for the pilot trial, an entirely separate sample was recruited (i.e., participants in the focus groups or individual interviews we excluded) to reduce bias and preserve independence in sampling.

## 2. Materials and Methods

### 2.1. Participants

Participant eligibility criteria included (1) a diagnosis of Stage III or IV lung cancer; (2) age 65 or older; (3) living at home; and (4) ability to speak and read in English. Exclusion criteria were (1) visual or hearing impairments that precluded participation and (2) severe, untreated mental illness that would preclude giving informed written consent. The eligibility criteria were the same for both the intervention development and pilot phases of the study.

### 2.2. Procedures

This study was approved by the Duke University Institutional Review Board (IRB number: Pro00102705; ClinicalTrials.gov: NCT04057196). Participants were purposively sampled and recruited from the Duke Cancer Institute in Durham, NC. The study team determined initial eligibility through electronic medical record review and subsequent research recruitment letters. Our clinical research coordinator then phoned eligible and interested participants to conduct a screening to determine their eligibility. All participants completed treatment sessions individually via telehealth, and tablets on loan were provided for their use (as needed). Assessments were completed at baseline, post-, and 1-month follow-up using REDCap [22]. Participants were paid USD 150 for their participation in the study, with USD 50 allocated for completing each of the three evaluations (baseline, post-intervention, and follow-up).

### 2.3. Measures

*Demographics.* Participants provided standard demographic information, including age, sex, race and ethnicity, marital status, income, and education.

*Feasibility and Acceptability*. We examined feasibility metrics related to the number of participants we could recruit during the study period, with a primary goal of 30 participants in 18 months or less, as well as adherence rates among those who received and completed the SST-LC intervention skills. We also examined participant satisfaction with the content and delivery of SST-LC as a metric for acceptability.

*Outcome Measures*. Participants completed self-report measures at baseline, post-treatment, and follow-up. The outcome measures are summarized below.

Psychological Distress. We measured distress using the Clinical Outcomes in Routine Evaluation 10 (CORE-10) [23]. The CORE-10 measure comprises ten questions about how participants have been feeling over the past week. Each item is rated on a 5-point Likert scale ranging from 0 (“not at all”) to 4 (“most or all of the time”), yielding total scores from 0 to 40 (higher scores reflect greater distress; sample item: “I have felt anxious or tense”). Psychometric evaluations in both clinical and nonclinical adult samples demonstrate high internal consistency, with Cronbach’s α = 0.90 overall; α = 0.92 in nonclinical; α = 0.94 in clinical) and test–retest reliability of ICC = 0.81 [23].

Depression. Depression was measured using the Beck Depression Inventory (BDI-II) [24,25]. The measure has 21 items. The BDI-II is scored by summing the ratings for the 21 items. Each item is rated on a 4-point Likert scale ranging from 0 to 3, producing total scores from 0 to 63 (higher scores indicate a greater level of depression; sample item: “I get as much satisfaction out of things as I used to”). The BDI-II has high internal consistency, with Cronbach’s α = 0.91 [11], and strong test–retest reliability (r = 0.93) [24].

Quality of Life and Well-being. We used the Functional Assessment of Cancer Therapy-Lung (FACT-L) [26] to assess the overall quality of life and dimensions of well-being. The FACT-L is a 36-item measure scored on a five-point Likert scale, ranging from 0 (not at all) to 4 (very much), that measures overall quality of life (QOL) across five dimensions: physical well-being, social/family well-being, emotional well-being, functional well-being, and a lung cancer subscale. A total FACT-L score is calculated by summing the five subscales, with scores ranging from 0 to 136. The higher the score, the better the QOL. Internal consistency of the five FACT-L subscales ranges from 0.56 to 0.89 [26].

Treatment Satisfaction. The Client Satisfaction Questionnaire (CSQ-8) [27] was used to assess satisfaction with care received at post-treatment. An overall score is calculated by summing the respondent’s rating (item rating) score for each scale item (ratings are from 1 to 4). For the CSQ-8 version, scores range from 8 to 32, with higher values indicating higher satisfaction [27]; we implemented a cut-off of 26 or above to indicate high satisfaction.

*SST-LC Session Content*. SST consists of four main phases, an orientation, exploration, adaptation, and maintenance plan phase. Sessions can be completed within 8 sessions or extended to 12 sessions, based on patient need and in collaboration with the interventionist. For participants completing 12 sessions, one additional session was provided in each of the four phases. Session content is summarized below.

*Sessions 1–3*. The *orientation* phase introduces the goals of SST in the context of advanced lung cancer. Interventionists build rapport with older adult patients by addressing emotional challenges and fears about living with lung cancer and how that has impacted their identity and how they see themselves. Behavioral activation skills are introduced (e.g., performing a pleasant activity to help reduce distress).

*Sessions 4–5.* The *exploration* phase consists of helping patients identify the self-discrepancies they are experiencing as they relate to living with cancer and discussing their own self-efficacy to manage their psychological distress and cancer. Personal strengths and existing coping strategies are reinforced. Self-evaluation skills, such as reflective exercises, are introduced to help patients actively reflect on their behaviors and emotional responses, increasing self-awareness and confidence in managing distress and how they cope.

*Sessions 6–7.* The *adaptation phase* consists of the patient and interventionist working together to develop goals (i.e., goal-setting skills) that are both prevention-focused and promotion-focused. Examples include committing to spending at least 30 min daily engaging in an activity that reinforces their key roles (e.g., being a parent or grandparent) to preserve identity. Create a weekly plan that includes specific actions/behaviors aligned with health (exercising, eating healthy) and how they can foster their independence. Practicing specific relaxation techniques to improve their focus, increase gratitude, and promote positive emotional experiences. Reaching out to one person from their support network each week to prevent feelings of isolation and burden. These skills are adapted and tailored to the needs of the older adult to address their current life circumstances, cognitive and physical limitations, and/or personality traits.

*Session 8.* For the *termination/maintenance plan phase*, each participant along with the interventionist, develops a maintenance plan that includes a list of daily strategies (e.g., self-monitoring forms), short-term goals (e.g., becoming more active in self-rewarding activities), and long-term goals.

### 2.4. Analysis

Focus group and individual interviews were analyzed using thematic content analysis [28] as described earlier. User testing was primarily descriptive in finalizing study procedures. For the pilot, our primary focus was to assess acceptability and feasibility metrics, including recruitment, adherence, and satisfaction with the intervention. Secondarily, descriptive statistics, normal distribution assumptions, and paired sample *t*-tests (with an alpha level of 0.05) were explored to examine potential changes in outcomes from pre-, post-, and follow-up assessments using SPSS Version 29 [29]. These tests were *strictly exploratory* given that pilot studies are typically underpowered and are designed primarily to assess feasibility and acceptability. In addition to exploring changes in outcomes, we also explored associated effect sizes with confidence intervals, and *p*-values.

Sample size considerations were informed by the consolidated standards of reporting trials (CONSORT) extension for pilot trials and by behavioral research in cancer supportive care [30,31,32,33,34,35,36,37]. Consistent with recommendations for pilot trial design a sample size of 30 participants was considered appropriate to address feasibility and acceptability.

## 3. Results

### 3.1. Demographics, Feasibility, and Acceptability Benchmarks for the Pilot Trial

**Figure 1:**
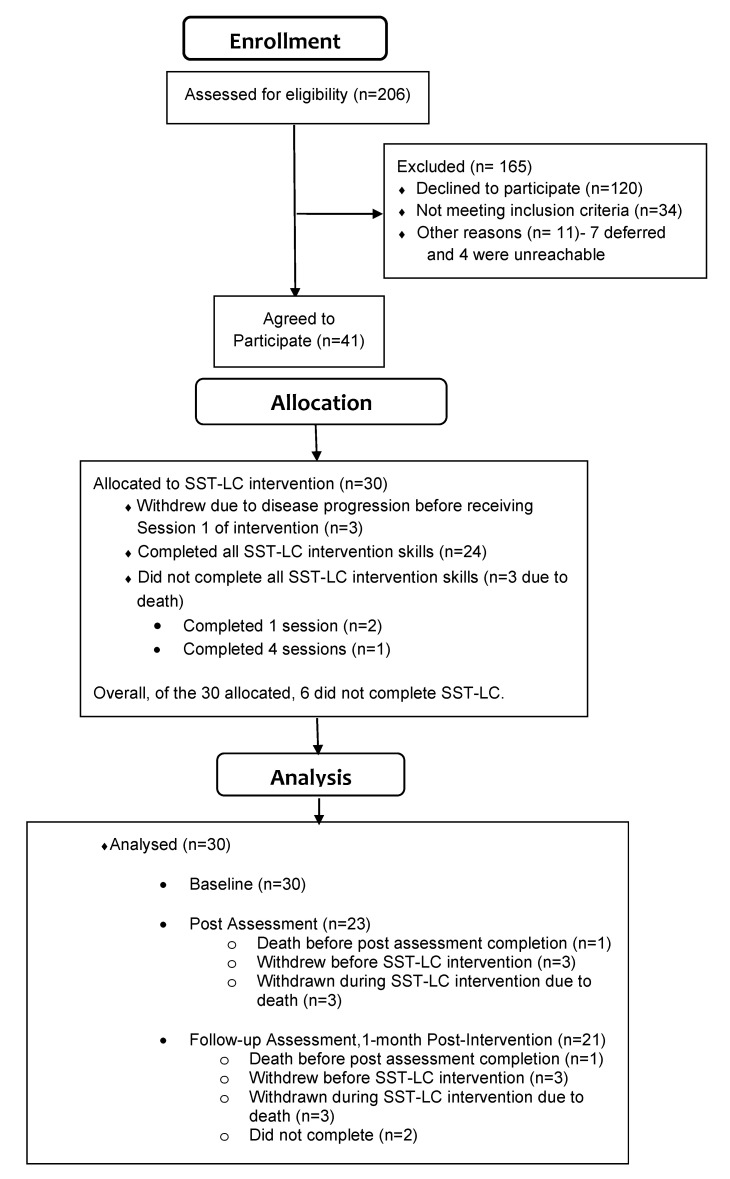
Study Flow CONSORT Diagram for Recruitment and Enrollment.

A total of 30 participants were recruited and enrolled in the study. See Figure 1 CONSORT diagram. Seventy-seven percent (23/30) of participants were female, with a mean age of 69.7 years old (range 65–83 years, SD = 4.95). Most participants were White/Caucasian (86.7%) or Black/African American (13.3%). See Table 1 for a complete summary of participant demographics.

We met our recruitment feasibility goal within two years for our pilot. For our secondary feasibility benchmark, we observed an excellent adherence rate of 89% (among those who received the intervention [N = 27], 24 completed/learned all session skills). Of these 24, 11 completed at least 8 sessions, and 13 participants completed 12 sessions. Most (85%) expressed high satisfaction (as reported in the CSQ-8) with the intervention at the conclusion of SST-LC (acceptability).

### 3.2. Exploration of Pre-to-Post Changes in Outcome Measures

From an exploratory basis, we examined the normal distribution of each outcome using the Shapiro–Wilk test. For outcomes that were normally distributed, we conducted our analysis using paired sample t-tests; for those that were not at specific time points (either at 12 or 16 weeks), we employed Wilcoxon signed-rank tests. Both approaches yielded the same conclusions. Given that the non-normally distributed outcomes did not have extreme outliers, and that parametric tests offer greater interpretability and consistency, we report our results with paired *t*-tests.

In examining *signal* of pre-post changes in distress, we found improvements in decreasing distress from baseline to 12 weeks post-intervention (*p* = 0.02); with improvements maintained at 1-month follow-up (*p* = 0.01). We also found improvements in depression (*p* = 0.001), emotional well-being (*p* = 0.05), functional well-being (*p* = 0.008), physical well-being (*p* = 0.11), social well-being (*p* = 0.53), and quality of life (*p* = 0.06) from baseline to 12 weeks post-intervention. See Table 2 and Table 3 for our results summary, including our results at follow-up.

We also invited participants to provide feedback following the intervention through brief exit interviews. Participants suggested reducing the total number of sessions (that during the trial ranged from 8 to 12) and shared that the SST intervention had a positive impact on their perceived resilience, as well as their ability to recover from cancer-related setbacks.

## 4. Discussion

Concomitant with physical health declines, several psychological and quality of life needs are prevalent in older adults with advanced lung cancer, such as increased depression, existential distress, uncertainty, and identity disruption [12,37,38]. As previously noted, available supportive-care interventions often overlook the aging concerns that older adults experience during this phase of life, and the need for tailored interventions, such as SST-LC, to address how their identity, role transitions, and health goals are impacted is paramount [12,13,14,15].

To the best of our knowledge, three previous studies tested interventions specifically designed to meet the unique needs of older adults living with advanced cancer. The first was a small-scale randomized control trial (RCT) that tested telephone-delivered cognitive-behavioral therapy (CBT) for older adults either undergoing active cancer treatment or within six months post-treatment (N = 29; 63% with advanced cancer) [39]. This study showed low acceptability with small effects on anxiety and no effects on depression. The second RCT focused on enhancing hope through film and hope-focused activities for patients with terminal cancer (N = 60), which improved hope and quality of life but did not change psychological distress [40]. The third study, a large-scale RCT of Dignity Therapy (N = 326), primarily involving older adults found improvements in quality of life but no changes in psychological distress [41]. Overall, these findings underscore a need for interventions designed for older adults to target for distress in this vulnerable population.

Our results extend traditional SST [18,19] to a novel context, highlighting the intervention’s versatility in mobilizing identity renegotiation, resilience-building, and pursuit of promotion and prevention goals. Among older adults with advanced lung cancer, SST-LC is well-suited to address discrepancies between former self-concepts and current physical/emotional realities, effectively supporting individuals as they gain a renewed sense of agency, adaptive coping skills, and resiliency in the face of uncertainty.

Specifically, our pilot evaluation of SST-LC suggested that this goal-focused supportive care intervention was both feasible and acceptable for older adults with advanced lung cancer. The brief nature of SST-LC and telehealth delivery likely increased participation, as evidenced by treatment adherence (89% completed all sessions), satisfaction (85% reported high satisfaction), and tailoring treatment content to address distress when living with advanced cancer. The use of the adaptome framework resulted in successful, iterative refinements of the intervention based on participant feedback [20]. We observed sustained improvements in psychological distress, depression, and functional well-being. These signals of preliminary efficacy persisted at the one-month follow-up, demonstrating the promise of SST-LC as a viable treatment in advanced cancer.

While this pilot study provides initial evidence that SST-LC has high feasibility, acceptability, and may reduce distress, we note specific priorities for future research. While a single-arm, small-sample design may be appropriate for testing feasibility and acceptability, it is underpowered to detect clinically significant effects [42]. The sample was predominantly White and English-speaking, which limits generalizability to representative populations. Additionally, conclusions about the sustainability of the intervention effects may require longer follow-up assessments. Finally, while telehealth delivery increases accessibility, it may hinder scalability in areas with limited technology support, particularly for those without internet access, which could prove challenging in situations with limited digital infrastructure.

## 5. Conclusions

Given that existing interventions often overlook age-specific and life-stage needs among older populations, such as identity disruption, uncertainty, and shifting goal priorities, SST-LC’s focus on resilience, goal pursuit, and identity management is invaluable for reducing distress and improving quality of life among older adults with advanced lung cancer. The extension of this work will require a larger randomized controlled trial, with a reduced number of sessions (e.g., 6), to assess whether SST for cancer populations compared to a control condition can relieve symptoms such as distress and improve quality of life, while also identifying potential mechanisms of the intervention (e.g., self-efficacy).

## Figures and Tables

**Table 1:** Demographic Characteristics of Pilot Trial Participants (N = 30).

Individual Characteristics		N	N%	Mean	Standard Deviation
Age				69.77	4.95
Gender	Male	7	23.3		
	Female	23	76.7		
Race	African American or Black	4	13.3		
	White	26	86.7		
Ethnicity	Not Hispanic	30	100.0		
	Hispanic	0	0.0		
Education	Less than high school	0	0.0		
	High school degree or GED	5	16.7		
	Some college or technical school	9	30.0		
	4-year college degree	6	20.0		
	Post-baccalaureate degree	10	33.3		
Marital	Married	21	91.3		
	Not married but living with a partner	2	8.7		
Income	Less than USD 20,000	3	10.3		
	USD 20,000–USD 39,999	3	10.3		
	USD 40,000–USD 59,999	8	27.6		
	USD 60,000–USD 79,999	4	13.8		
	USD 80,000–USD 99,999	6	20.7		
	USD 100,000–USD 120,999	3	10.3		
	USD 121,000 or more	2	6.9		
Children under the age of 18	No	29	96.7		
	Yes	1	3.3		

**Table 2:** SST-LC Pilot Trial Summary of Outcome Changes from Baseline to 12 Weeks.

	Mean (*SD*)		Baseline to 12 Weeks Post-Intervention (N = 23)				
Outcome Variable	Baseline	Post-Treatment	t	*df*	*p*	Cohen’s *d*	95% CI for *d* [Lower, Upper Bound]
Distress	14.55 (3.93)	12.67 (3.62)	2.46	22	0.022 *	0.51	[0.07, 0.94]
Depression	9.41 (6.09)	6.17 (6.07)	3.63	22	0.001 *	0.76	[0.28, 1.2]
*FACT* *Dimensions*							
Emotional Well-being	17.83 (3.89)	18.65 (2.95)	−2.04	22	0.05 *	−0.43	[−0.849, 0.006]
Physical Health - Functional Well-Being	18.04 (5.18)	21.41 (5.08)	−2.89	22	0.008 *	−0.60	[−1.04, −0.15]
Physical Health - Physical Well-Being	23.03 (4.08)	24.30 (3.81)	−1.67	22	0.11	−0.35	[−0.77, 0.08]
Social Well-Being	23.09 (5.66)	23.66 (3.48)	0.64	22	0.53	0.13	[−0.28, 0.54]
Quality of Life	102.22 (16.71)	109.12 (16.0)	−1.95	22	0.06	−0.41	[−0.83, 0.02]

Note. Results from these paired sample t-tests are exploratory. Cohen’s d: small effect = 0.20; medium effect = 0.50; large effect = 0.80. CI = Confidence Interval. * p < 0.05.

**Table 3:** SST-LC Pilot Trial Summary of Outcome Changes from Baseline to 16 Weeks.

	Mean (*SD*)		Baseline to 16 Weeks Post-Intervention (N = 21)				
Outcome Variable	Baseline	Follow-Up	t	*df*	*p*	Cohen’s *d*	95% CI for *d* [Lower, Upper Bound]
Distress	14.55 (3.93)	12.30 (3.60)	2.74	20	0.013 *	0.59	[0.13, 1.06]
Depression	9.41 (6.09)	6.13 (5.10)	3.27	20	0.004 *	0.71	[0.23, 1.19]
*FACT* *Dimensions*							
Emotional Well-being	17.83 (3.89)	19.48 (2.94)	−2.90	20	0.009 *	−0.63	[−1.10, −0.16]
Physical Health - Functional Well-Being	18.04 (5.18)	19.75 (5.62)	−1.21	20	0.24	−0.26	[−0.69, 0.17]
Physical Health - Physical Well-Being	23.03 (4.08)	22.94 (4.57)	0.92	20	0.37	0.20	[−0.23, 0.63]
Social Well-Being	23.09 (5.66)	23.07 (4.03)	1.30	20	0.21	0.28	[−0.16, 0.72]
Quality of Life	102.22 (16.71)	106.30 (17.51)	−0.46	20	0.65	−0.10	[−0.53, 0.33]

Note. Results from these paired sample t-tests are exploratory. Cohen’s d: small effect = 0.20; medium effect = 0.50; large effect = 0.80. CI = Confidence Interval. * p < 0.05.

## Data Availability

Our data are available upon request pending approvals from our Institutional Review Board. If interested, please e-mail the corresponding author.

## Abbreviations

The following abbreviations are used in this manuscript:

SST	Self-System Therapy
SST-LC	Self-System Therapy for Lung Cancer
QOL	Quality of Life
FACT-L	Functional Assessment of Cancer Therapy-Lung
CORE-10	Clinical Outcomes in Routine Evaluation-10
BDI-II	Beck Depression Inventory
CSQ-8	The Client Satisfaction Questionnaire-8
ICC	Intraclass correlation coefficient
